# Dense Molten Salt Method for Large-Scale Synthesis of Rare Earth Hafnate Powders

**DOI:** 10.3390/ma19091765

**Published:** 2026-04-26

**Authors:** Zhijun Xiao, Yongxin Wang, Jingjie Li, Zhong Huang, Yu Zhang, Shaowei Zhang

**Affiliations:** 1 State Key Laboratory of Advanced Refractories, Wuhan University of Science and Technology, Wuhan 430081, China; xiaozhijun@wust.edu.cn (Z.X.); wangyongxin@mails.neu.edu.cn (Y.W.); 2 Joint International Research Laboratory of Refractories and Metallurgy, Wuhan University of Science and Technology, Wuhan 430081, China; 3 The Cultivation Base of Shanxi Key Laboratory of Mining Area Ecological Restoration and Solid Wastes Utilization, Shanxi Institute of Technology, Yangquan 045000, China; zhangyuiwh@whu.edu.cn; 4 College of Engineering, Mathematics and Physical Sciences, University of Exeter, Exeter EX4 4QF, UK; s.zhang@exeter.ac.uk

**Keywords:** RE_2_Hf_2_O_7_, dense molten salt, salt pools, hafnate, large-scale synthesis

## Abstract

Rare earth hafnates (RE_2_Hf_2_O_7_; RE = La, Gd, Nd, Eu, etc.) with low thermal conductivity and excellent high-temperature stability are indispensable key materials in extreme environments and high-tech fields. In this work, a dense molten salt method (DMS) was developed for mass preparation of hafnate powders including La_2_Hf_2_O_7_, Nd_2_Hf_2_O_7_, Gd_2_Hf_2_O_7_, Eu_2_Hf_2_O_7_, and even (La_0.2_Nd_0.2_Sm_0.2_Eu_0.2_Gd_0.2_)_2_Hf_2_O_7_, which only allowed the trace salt volatilization, while the internal salt “micro-pools” significantly promoted the in-situ formation of target products at relatively lower temperatures. Using La_2_Hf_2_O_7_ as an example, it could be successfully prepared at 1100 °C with 1:1 mass ratio of salt to reactant, both of which are much lower than those of traditional “powdery” molten salt method. Furthermore, only ~6 wt.% of salt loss was detected in current dense route, while it was as high as ~80 wt.% in the traditional one. A large-scale synthesis of RE_2_Hf_2_O_7_ powder by DMS may be achievable by stacking these dense blocks in a tunnel kiln, suggesting its potential applicability and scalability toward industrial production.

## 1. Introduction

Rare earth hafnate with the pyrochlore structure (general formula RE_2_Hf_2_O_7_) is a class of key materials that are indispensable in extreme environments (ultra-high temperature, strong corrosion and radiation) and high-tech fields (nuclear energy, aerospace, and microelectronics) due to their high melting point, excellent thermal and chemical stability [1,2,3,4,5,6,7,8]. Currently, common preparation methods for rare earth hafnate mainly include high-temperature solid state reaction, co-precipitation, and combustion syntheses [9,10,11]. For example, Vlášková et al. prepared the Er_2_Ir_2_O_7_ powder at 1200 °C for 12 h by a solid state reaction [12]. Ji et al. synthesized the La_2_Hf_2_O_7_ powder using glycine or EDTA as a fuel through a combustion route [13]. Balencie et al. synthesized HfGeO_4_ by using a co-precipitation process at 1100 °C for 6 h [14]. Despite the achievements of these methods, they typically require high temperature or prolonged reaction duration, additional chemical reagents and complex equipment, which are unfavorable for large-scale production. Therefore, it is crucial to develop an efficient method for the preparation of RE_2_Hf_2_O_7_.

Molten salt method employs the low-melting-point salts as a reaction medium [15,16,17], which can significantly reduce the reaction temperature (typically by 300–500 °C compared to the solid-state methods), while promoting the ion diffusion and mass transfer, enabling the rapid preparation of pure-phase powders with small particle size and high crystallinity. Pokhrel et al. [18] prepared RE_2_Hf_2_O_7_ (RE = La and Pr) nanoparticles in molten salt at 650 °C using the complex hydroxide precursor as raw materials. In our previous works, Huang et al. [19] prepared a pyrochlore-type La_2_Zr_2_O_7_ at 1100 °C for 3 h. Xue et al. [20] prepared a pure-phase (Mg_0.2_Co_0.2_Ni_0.2_Cu_0.2_Zn_0.2_)O at 850 °C with tunable structures including rock salt, spinel and pyrochlore. However, it is widely recognized that the molten salt method would be difficult to use for industrial production, because of the inevitable volatilization of salt during high-temperature treatment, which not only easily corrodes the equipment, but also results in the use of a large quantity of salt to maintain the necessary liquid phase condition [21,22,23]. This key drawback leads to higher costs and greater operational difficulties for molten salt method, making it difficult to transfer from the lab synthesis to the actual industrial production.

**Figure 1:**
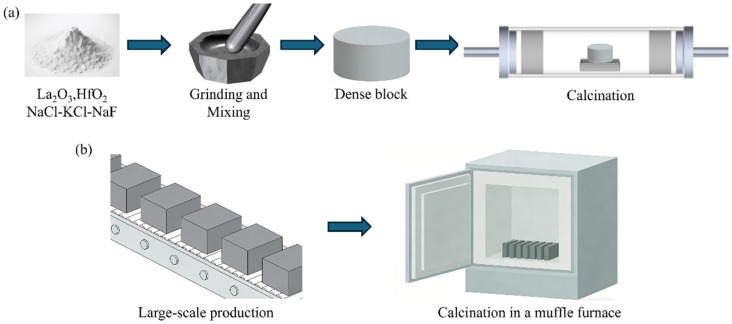
(**a**) Schematic diagram of the preparation of rare earth hafnate powders by DMS. (**b**) Mass preparation of rare earth hafnate by DMS.

Herein, we propose a dense molten salt synthesis (DMS), which involves pressing powdered raw materials into a dense block, promoting intimate contact between reactant particles, thereby reducing the amount of salt required to sustain the reaction environment (Figure 1a). By using La_2_O_3_ and HfO_2_ as raw materials and an NaCl-KCl-NaF mixed salt as molten salt, we successfully synthesized the pure-phase La_2_Hf_2_O_7_ powder at 1100 °C, and the salt volatilization was simultaneously restricted within the dense block, mitigating the equipment corrosion during calcination. DMS could retain the advantages of conventional molten salt synthesis (CMS), while further improving the preparation efficiency by reducing salt usage and volatilization. To demonstrate the extensibility of DMS, other powders of RE_2_Hf_2_O_7_ (RE = Gd, Nd, and Eu) and even (La_0.2_Nd_0.2_Sm_0.2_Eu_0.2_Gd_0.2_)_2_Hf_2_O_7_ could also be prepared, which could potentially enable a mass production by stacking these blocks in an industrial furnace (Figure 1b). These findings suggest the application potential of DMS for scale-up synthesis of a series of ceramic powders.

## 2. Experimental

### 2.1. Preparation of La_2_Hf_2_O_7_ Powder

The flowchart of DMS process is presented in Figure 1a. In brief, the raw material powders (La_2_O_3_, HfO_2_ and NaCl-KCl-NaF) were manually mixed in an agate mortar for 30 min to obtain a homogeneous mixture. Then, a certain mass of the mixed powder was pressed into dense blocks with sizes of Φ20 mm, Φ30 mm, Φ50 mm and 140 mm × 25 mm × 18 mm using a manual hydraulic press set at 50 MPa (holding time of 3 min). The calcination process was carried out in a tube furnace: the temperature was first increased to 1000 °C at a heating rate of 5 °C/min, then further increased to 1100 °C at 3 °C/min, followed by a dwell time of 2 h at 1100 °C. After cooling, the blocks were crushed, washed with deionized water and then vacuum-filtered until most of the residual salts were removed, further purification can be adopted for more demanding applications. Finally, the final powders were vacuum-dried overnight at 60 °C for further characterizations. The preparation methods for other rare earth hafnates and high-entropy hafnate powders were the same, and the detailed stoichiometric ratio and preparation conditions are listed in Table S1.

### 2.2. Characterization

The crystal structures of the samples were measured using a powder X-ray diffractometer (X’ pert pro, Philips, Amsterdam, The Netherlands) at 40 kV and 40 mA using Cu Kα radiation (λ = 0.1542 nm). ICDD cards of 73-0445, 78-0049, 83-1345, 24-0425, 24-0078 and 24-0410 were used to reveal the patterns of La_2_Hf_2_O_7_, HfO_2_, La_2_O_3_, Gd_2_Hf_2_O_7_, Nd_2_Hf_2_O_7_ and Eu_2_Hf_2_O_7_ respectively. The microstructure of the sample was characterized using a ThermoFisher Helios 5 CX DualBeam (SEM, Thermo Fisher Scientific, Waltham, MA, USA) and a high-resolution transmission electron microscope (HRTEM, JEM-2100UHRS, JEOL, Tokyo, Japan, operated at 200 kV). The chemical composition was determined by Oxford UltimMax 170 EDS(Oxford Instruments, Abingdon, UK).

## 3. Results and Discussion

### 3.1. Preparation of La_2_Hf_2_O_7_ Powder via DMS

**Figure 2:**
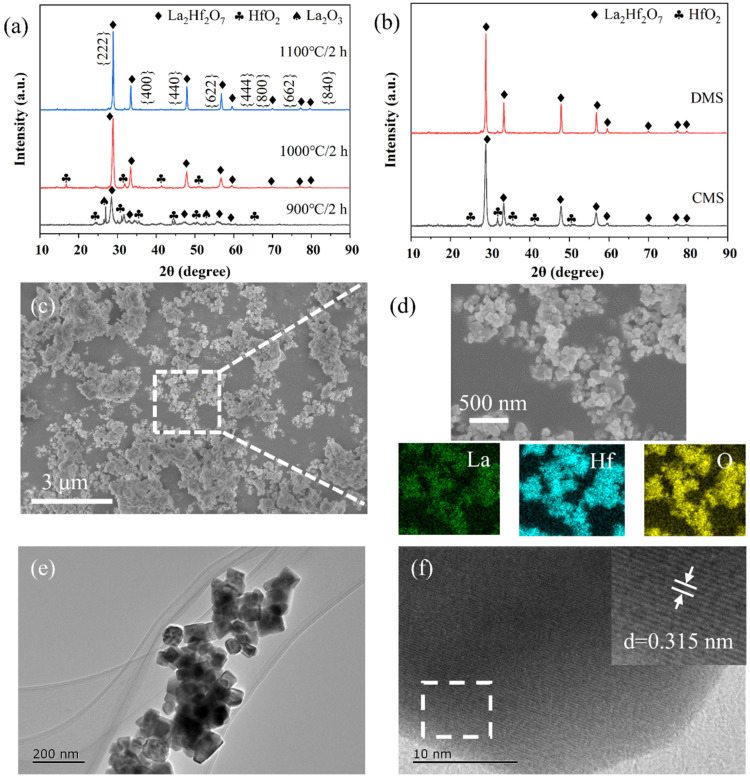
(**a**) XRD patterns of as-prepared samples at different temperatures by DMS. (**b**) XRD patterns of samples prepared by DMS and CMS methods. (**c**) SEM, (**d**) EDS-mapping, (**e**) TEM and (**f**) HRTEM images of La_2_Hf_2_O_7_ powder prepared by DMS at 1100 °C.

Figure 2a shows the XRD patterns of La_2_Hf_2_O_7_ powders prepared using DMS at different temperatures. It could be seen that the sample calcined at 900 °C contained the phases of unreacted La_2_O_3_ and HfO_2_. As the temperature rose to 1000 °C, the reaction proceeded more thoroughly, and only a small amount of raw materials were observed, and La_2_Hf_2_O_7_ became the predominant component. When the temperature was further increased to 1100 °C, only La_2_Hf_2_O_7_ phase was detected in the product, indicating a complete reaction between La_2_O_3_ and HfO_2_. It should be noted that a holding time of 2 h was used for all conditions described above. When the holding time at 1100 °C was reduced to 1 h, unreacted raw materials were detected, indicating a requirement of at least 2 h holding time (Figure S1). In order to highlight the advantage of the current method, we carried out the synthesis using the conventional molten salt method (CMS) under identical conditions, which means the same masses of salt and reactants were used. The only difference was that, in the CMS, the raw materials were not pressed into a dense block; instead, the homogeneously mixed raw material powder was placed directly into a crucible and calcined at the same temperature and for the same reaction time. The XRD patterns in Figure 2b show that initial raw materials with a “powdery” form cannot be used to synthesize pure-phase La_2_Hf_2_O_7_. The SEM image reveals that the as-obtained La_2_Hf_2_O_7_ powder possessed a distinct pyrochlore-type octahedral structure, and the particle size ranged from several tens to hundreds of nanometers (Figure S2). Furthermore, EDS-mapping results demonstrate a uniform spatial distribution of the constituent elements (La, Hf, and O), indicating a homogeneous chemical composition at the microscale (Figure 2c,d).

To further investigate the structural characteristics of La_2_Hf_2_O_7_, TEM and HRTEM analyses were performed, as shown in Figure 2e,f. It shows that the La_2_Hf_2_O_7_ particles had a size in the nanometer range. The HRTEM image in Figure 2f reveals the lattice fringes with a spacing of approximately 0.315 nm, which is in agreement with the standard d-spacing for (222) plane of pyrochlore phase (ICDD No. 01-073-0445; d(222) = 0.312 nm) [24,25], indicating a high crystallinity of as-prepared powder.

### 3.2. Synthesis Mechanism of DMS

**Figure 3:**
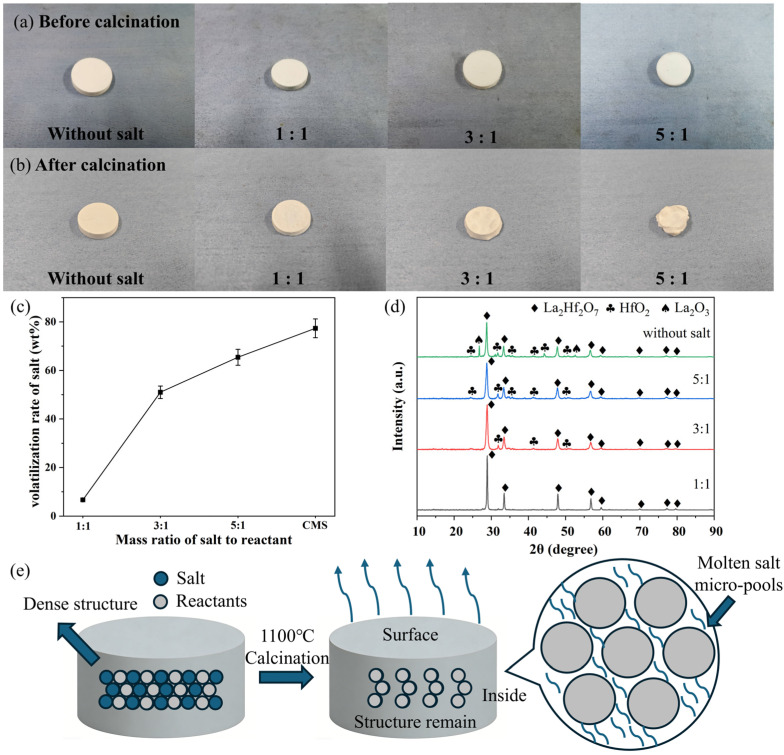
(**a**,**b**) Photos of dense blocks with different mass ratios of salt to reactant before and after calcination, and (**c**) the corresponding salt volatilization rates, and (**d**) XRD patterns of as-prepared La_2_Hf_2_O_7_. (**e**) Synthesis mechanism diagram of DMS.

In order to clarify the synthesis mechanism of DMS, the Φ20 mm blocks with different salt contents were calcined at 1100 °C for 2 h, and their appearances are shown in Figure 3a,b. In the case of samples without salt and with 1:1 mass ratio of salt to reactants, whether calcined or not, the dense blocks remained essentially unchanged, and a salt volatilization rate of only about 6 wt.% was detected in the latter case (Figure 3c). The volatilization rate was calculated by thrice measuring the mass difference of the samples before and after calcination, which formula is “(Mass_initial_ − Mass_final_)/Mass_salt-initial_ × 100%”. When the salt ratio was increased to 3:1, the blocks shrank after calcination, and the bulges and cavities appeared on the surface, which should be formed by salt aggregation and subsequent volatilization when the temperature was over the melting point of NaCl-KCl-NaF (604.1 °C [26]). As a result, the corresponding salt volatilization rate was high up to 50 wt.%. At a salt-to-reactant mass ratio of 5:1, the blocks underwent a significant irregular shrinkage after calcination, and the salt volatilization rate was further increased to 60 wt.%, indicating that the higher the molten salt content, the greater the amount of salt volatilization.

The corresponding XRD patterns are presented in Figure 3d. In the absence of salt, although the block appearance remained unchanged, some of the starting La_2_O_3_ and HfO_2_ were present in the product. For the sample with 1:1 mass ratio, the block was similar to that without salt, but it consisted of pure-phase La_2_Hf_2_O_7_, indicating that the molten salt could significantly promote the reaction between La_2_O_3_ and HfO_2_. In comparison, when the salt ratios were 3:1 and 5:1, the peaks corresponding to La_2_O_3_ and HfO_2_ were unexpectedly detected again, which became even more pronounced at a higher mass ratio. Combining this with the aforementioned result, it suggests two key points of the current DMS: (1) it is generally believed that the larger the amount of molten salt used, the better for powder synthesis in CMS process [27]. However, this is not the case in DMS; (2) preservation of block shape would help reduce the volatilization of molten salt.

Based on the above results and related literature [28], Figure 3e suggests a possible synthesis process of DMS. The uniformly mixed salt and starting powders are pressed into a dense block, ensuring the close contact between particles. As the temperature rises to the salt’s melting point (604.1 °C), the salt begins to melt to form the liquid reaction environment. When the salt content is moderate (1:1 mass ratio), the limited molten salt is unlikely to disrupt the dense structure. As demonstrated in Figure S5, the density of the block even increased with porosity decreasing from 33% to 24%, which is considered to generate strong physical confinement effect, that may effectively reduce the diffusion channels for salt. Instead, the salts are believed to form numerous micro-pools at the interfaces between La_2_O_3_ and HfO_2_ particles, which may promote the mass transfer and diffusion (as indicated in Figures S6 and S7), thereby possibly enhancing the reaction kinetics of La_2_Hf_2_O_7_ formation [29]. Since most of these molten micro-pools are located inside the dense block, only a small amount of surface salt appears to be volatilized. If the salt content is too high (3:1 and 5:1 mass ratios), the block structure is likely to collapse because of salt melting. However, this temperature is still far below the synthesis temperature of La_2_Hf_2_O_7_ (1250 °C [30]), and during this heating process, a large amount of molten salt evaporates instead. This not only leads to an incomplete reaction, but also results in waste of molten salt and potential risk of equipment corrosion. In summary, DMS is suggested to modify the reaction medium from a large molten salt bath (generally formed in CMS) to the microzones within a dense block, reducing both the required amount of salt and its volatilization during the heating process.

### 3.3. Scalability of DMS

**Figure 4:**
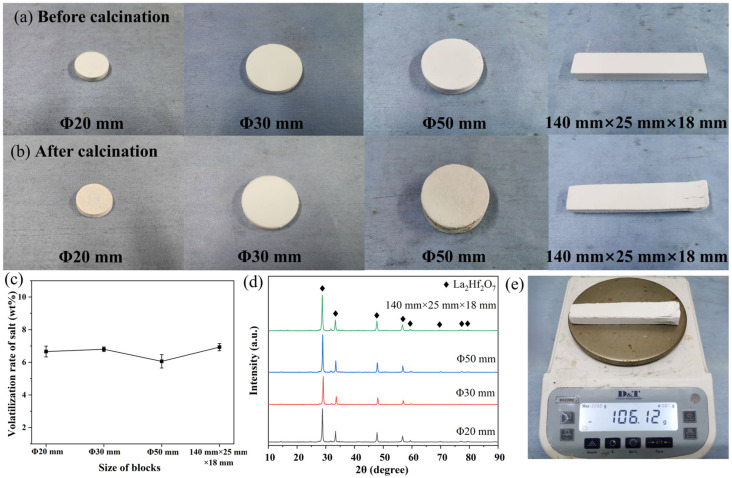
(**a**,**b**) Photos of dense blocks with different sizes before and after calcination, and the corresponding (**c**) salt volatilization rates, and (**d**) XRD patterns of as-prepared La_2_Hf_2_O_7_. (**e**) Weight of the sample with size of 140 mm × 25 mm × 18 mm before the residual salts were removed.

Based on the “block” form of DMS, it has the potential to be applied for scale-up production. Figure 4a,b show the appearances of dense blocks with sizes of Φ20 mm, Φ30 mm, Φ50 mm, and 140 mm × 25 mm × 18 mm before and after calcination (1100 °C/2 h, 1:1 mass ratio of salt to reactant). It can be observed that these block samples remained well-preserved with a minor dimensional shrinkage. The salt volatilization rates were all around 6.0 wt.% no matter the sizes of blocks (Figure 4c), and the calcined block had sufficient strength to be removed from the crucible (Figure S3). Analysis of the corresponding XRD patterns indicates that the block size did not affect the product purity, and pure-phase La_2_Hf_2_O_7_ could be observed (Figure 4d). Notably, one block with size of 140 mm × 25 mm × 18 mm could achieve the synthesis of 56.55 g La_2_Hf_2_O_7_ powders after removing the residual salts (Figure 4e). Based on these results, it is reasonable to infer that DMS holds promise for large-scale production. On the one hand, these dense blocks of DMS could be directly stacked on an industrial kiln, and calcined without complicated operations, as shown in Figure 1b. On the other hand, the low salt usage and volatilization of DMS can reduce raw material costs, while minimizing salt corrosion on equipment, aligning well with the industrial demands for low cost and high efficiency.

**Figure 5:**
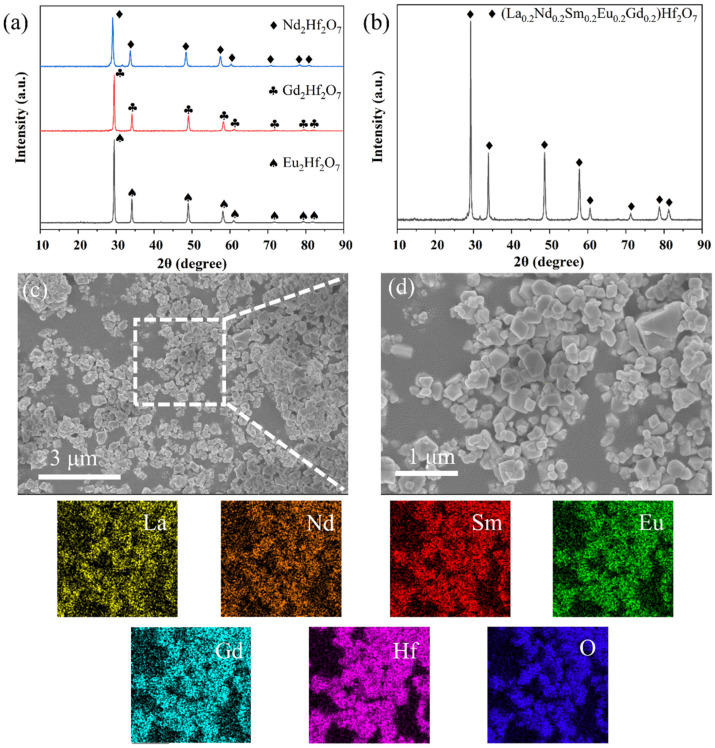
(**a**) XRD patterns of as-prepared RE_2_Hf_2_O_7_ (RE = Gd, Nd, Eu), and (**b**) (La_0.2_Nd_0.2_Sm_0.2_Eu_0.2_Gd_0.2_)_2_Hf_2_O_7_ (JCPDS No. 73-0445, 24-0425, 24-0778, 24-0078 and 24-0410) powders, and (**c**,**d**) the corresponding SEM and EDS-mapping of the high-entropy sample.

To verify the applicability of DMS for other rare earth hafnates and even high-entropy one, RE_2_Hf_2_O_7_ (RE = Gd, Nd and, Eu) and (La_0.2_Nd_0.2_Sm_0.2_Eu_0.2_Gd_0.2_)_2_Hf_2_O_7_ powders were prepared under an identical condition (block size of Φ20 mm, 1100 °C/2 h, and 1:1 mass ratio of salt-to-reactant). The XRD patterns in Figure 5a,b indicate that all these rare earth hafnates were pure-phases with high peak intensities. The SEM images reveal that the high-entropy hafnate powders exhibit a polyhedral structure, and the particle size was about several hundred nanometers (Figure S4). The EDS-mapping shows a homogeneous distribution of elements (Figure 5c,d). These characteristics align with those of products reported in other literature [31,32,33], confirming that the DMS method would also be applicable to other ceramic powders. Table 1 provides a comparison of synthesis conditions for various rare earth pyrochlore systems (including hafnates, zirconates, and cerates) reported in the literature. It can be seen that DMS requires a comparable reaction temperature and a shorter holding time, further verifying the scalability of this work.

## 4. Conclusions

In summary, we demonstrate that DMS may be scalable for the large-scale production of rare earth hafnate powders. It could enable an efficient synthesis of phase-pure RE_2_Hf_2_O_7_ at reduced temperatures, owing to the confined molten salt “micro-pools” within the dense blocks, while suppressing the salt volatilization with a lower salt usage. The DMS exhibits a broad applicability across various RE_2_Hf_2_O_7_ and high-entropy hafnate, and all these hafnates could be successfully prepared at 1100 °C for 2 h with a 1:1 mass ratio of salt to reactant. A dense La_2_Hf_2_O_7_ block with size of 140 mm × 25 mm × 18 mm was prepared, with a product weight of ~56.55 g per batch in the lab. This approach allows for a large-scale production by simply accommodating these blocks in conventional kilns, offering a practical and cost-effective route for industrial production of advanced ceramic powders.

## Figures and Tables

**Table 1:** Comparison of synthesis conditions for RE_2_Hf_2_O_7_ powders and related pyrochlore systems (zirconates and cerates) reported in the literature.

Materials	Synthesis Method	Synthesis Temperature (°C)	Holding Time (h)	Reference
La_2_Hf_2_O_7_	Co-precipitation	>1000	6	[18]
La_2_Zr_2_O_7_	Sol–gel	1000	6	[34]
La_2_Zr_2_O_7_	Molten salt	1100	3	[18]
La_2_Ce_2_O_7_	Molten salt	1100	3	[21]
Gd_2_Hf_2_O_7_	Solid state	1400	4	[35]
Eu_2_Hf_2_O_7_	Solid state	1400	4	[35]
(La_0.2_Nd_0.2_Sm_0.2_Eu_0.2_Gd_0.2_)_2_Hf_2_O_7_	Solid state	1600	10	[5]
RE_2_Hf_2_O_7_ (RE = La,Gd,Nd,Eu), (La_0.2_Nd_0.2_Sm_0.2_Eu_0.2_Gd_0.2_)_2_Hf_2_O_7_	Dense molten salt	1100	2	This work

## Data Availability

The original contributions presented in this study are included in the article/Supplementary Material. Further inquiries can be directed to the corresponding authors.

## Supplementary Materials

The following supporting information can be downloaded at: https://www.mdpi.com/article/10.3390/ma19091765/s1, Figure S1: XRD patterns of as-prepared samples with different holding time; Figure S2: Particle size distribution of as-prepared La_2_Hf_2_O_7_ powder; Figure S3: Appearance of the calcined block with size of 140 mm × 25 mm × 18 mm; Figure S4: Particle size distribution of as-prepared (La_0.2_Nd_0.2_Sm_0.2_Eu_0.2_Gd_0.2_)_2_Hf_2_O_7_ powder; Figure S5: Porosities and bulk densities of the block samples (Φ20 mm, mass ratio of salt to reactants was 1:1) before and after calcination at 1100 °C by DMS; Figure S6: (a) SEM images of the fracture surfaces of the block sample (Φ20 mm, mass ratio of salt to reactants was 1:1) before calcination, and (b) the corresponding EDS-mapping; Figure S7: (a) SEM images of the fracture surfaces of the block sample (Φ20 mm, mass ratio of salt to reactants was 1:1) after calcination, and (b) the corresponding EDS-mapping; Table S1: Processing parameters of DMS for RE_2_Hf_2_O_7_ (RE = La, Gd, Nd, Eu) and (La_0.2_Nd_0.2_Sm_0.2_Eu_0.2_Gd_0.2_)_2_Hf_2_O_7_ powders.
