# Supplementary material for: Dense Molten Salt Method for Large-Scale Synthesis of Rare Earth Hafnate Powders
Source: Materials (Basel). 2026 Apr 26;19(9):1765. doi: 10.3390/ma19091765 (PMC13165362; doi:10.3390/ma19091765)
Supplement: Supplementary file 1 [file materials-19-01765-s001.zip › materials-4116604-supplementary.pdf]

Supplementary Information

Dense Molten Salt Method for Large-Scale Synthesis of Rare Earth Hafnate Powders

Zhijun Xiao [1], Yongxin Wang [1], Jingjie Li [1,2,*], Zhong Huang [1,2,*], Yu Zhang [3] and Shaowei Zhang [4]

[1] State Key Laboratory of Advanced Refractories, Wuhan University of Science and Technology, Wuhan 430081, China
[2] Joint International Research Laboratory of Refractories and Metallurgy, Wuhan University of Science and Technology, Wuhan 430081, China
[3] The Cultivation Base of Shanxi Key Laboratory of Mining Area Ecological Restoration and Solid Wastes Utilization, Shanxi Institute of Technology, Yangquan 045000, China
[4] College of Engineering, Mathematics and Physical Sciences, University of Exeter, Exeter EX4 4QF, UK
* Correspondence: lijingjie@wust.edu.cn (J.L.); huangzhong@wust.edu.cn (Z.H.)

Raw materials

Rare earth oxides (La_2O_3, Nd_2O_3, Sm_2O_3, Eu_2O_3, Gd_2O_3,), HfO_2, (99.9% purity), were purchased from Aladdin Biochemical Technology Co. Ltd, Shanghai, (China). NaCl, KCl and NaF (99.9% purity) were purchased from Sinopharm Chemical Reagent Co. Ltd, (China). Deionized water was used as the solvent to remove the salt after calcination.

Characterization of the block samples

The relative density (ρ_2) of block sample was measured by the Archimedes method, which is calculated using the following equations:

$$\rho = \frac{m_0 \times \rho_1}{m_1 - m_2} \quad [1]$$

$$\rho_2 = \frac{\rho}{\rho_0} \times 100\% \quad [2]$$

Where, ρ is the apparent density of the sample, ρ_1 the density of distilled ethanol at room temperature, m_2 the suspended weight of the sample after boiling in ethanol for 30 min, m_1 the weight of the wet sample, m_0 the weight of the dried sample, and ρ_0 the true density determind by the fully automatic true density analyzer. All the tests were repeated three times.

Table S1. Processing parameters of DMS for $Re_2Hf_2O_7$ (Re=La, Gd, Nd, Eu) and $(La_{0.2}Nd_{0.2}Sm_{0.2}Eu_{0.2}Gd_{0.2})_2Hf_2O_7$ powders.

Products	Reactants	Salts	Molar Ratio (Reactants)	Temperature (°C)	Holding time (h)	Mass Ratio (salt to reactants)	Block size (mm)
$La_2Hf_2O_7$	La_2O_3/HfO_2	NaCl-KCl-NaF (45.6 wt%-43.5 wt%-10.9 wt%)	1:2	900	2	1:1	Φ20
				1000	2	1:1	Φ20
				1100	1	1:1	Φ20
					2	1:1	Φ20
						3:1	
						5:1	
						1:1	Φ30
							Φ50
							140×25×18
$Nd_2Hf_2O_7$	Nd_2O_3/HfO_2				2	1:1	Φ20
$Gd_2Hf_2O_7$	Gd_2O_3/HfO_2				2	1:1	Φ20
$Eu_2Hf_2O_7$	Eu_2O_3/HfO_2				2	1:1	Φ20
$(La_{0.2}Nd_{0.2}Sm_{0.2}Eu_{0.2}Gd_{0.2})_2Hf_2O_7$	$La_2O_3/Nd_2O_3/Sm_2O_3/Eu_2O_3/Gd_2O_3/HfO_2$		1:1:1:1:1:10		2	1:1	Φ20

Note: (1).The absolute masses of one block are as follows: 3 g for Φ20 mm, 7 g for Φ30 mm, 85 g for Φ50 mm, and 110 g for the 140 mm × 25 mm × 18 mm block.

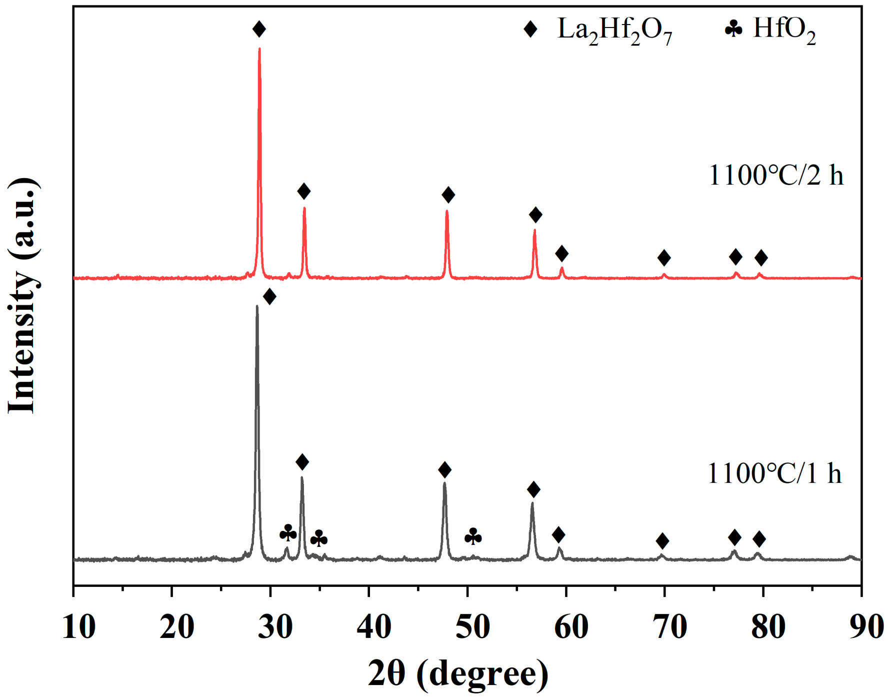

Figure S1. XRD patterns of as-prepared samples with different holding time.

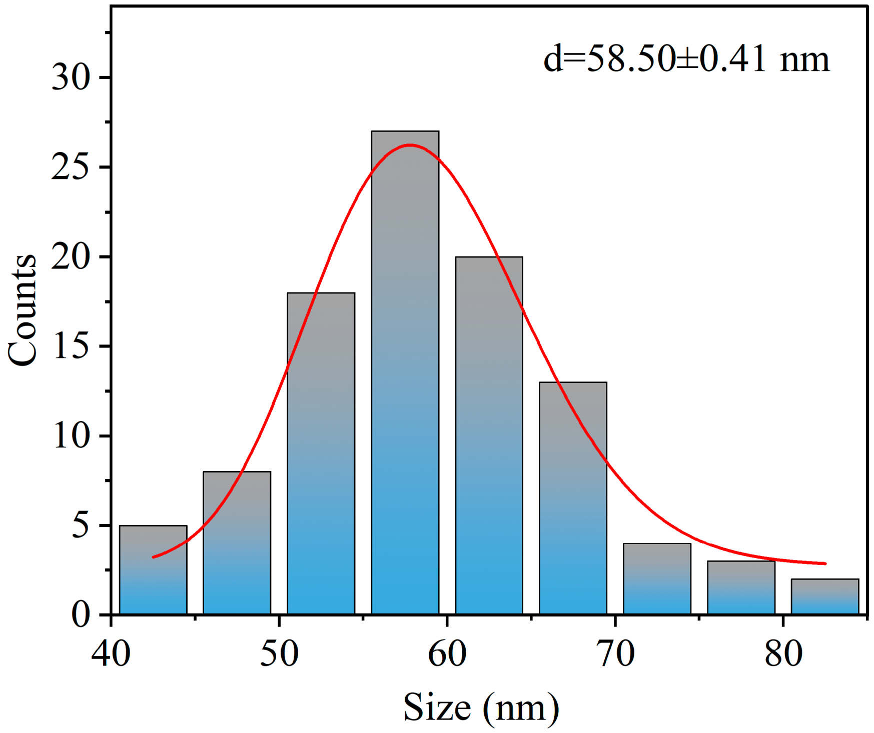

Figure S2. Particle size distribution of as-prepared $La_2Hf_2O_7$ powder.

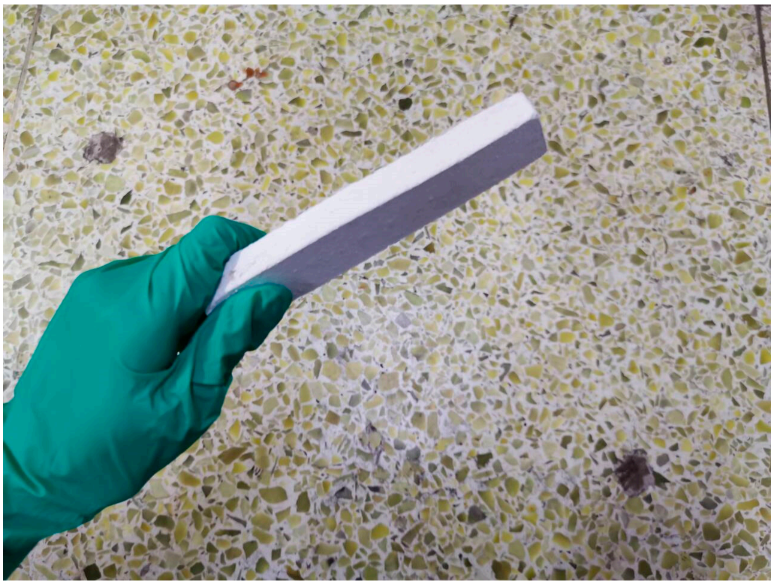

Figure S3. Appearance of the calcined block with size of 140 mm×25 mm×18 mm.

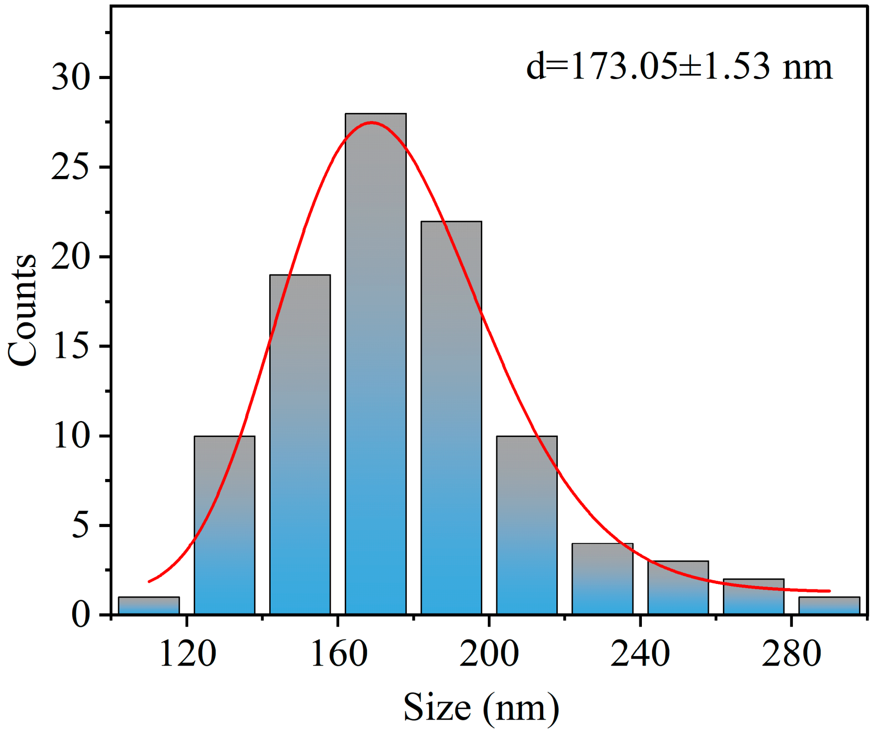

Figure S4. Particle size distribution of as-prepared $(La_{0.2}Nd_{0.2}Sm_{0.2}Eu_{0.2}Gd_{0.2})_2Hf_2O_7$ powder.

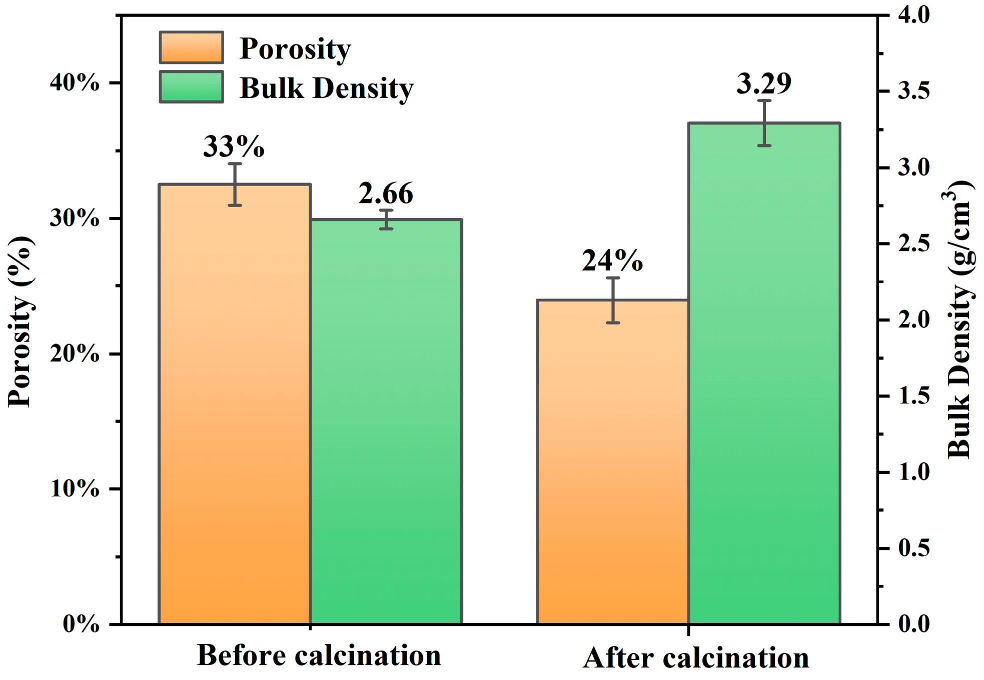

Figure S5. Porosities and bulk densities of the block samples (Φ20 mm, mass ratio of salt to reactants was 1:1) before and after calcination at 1100 °C by DMS.

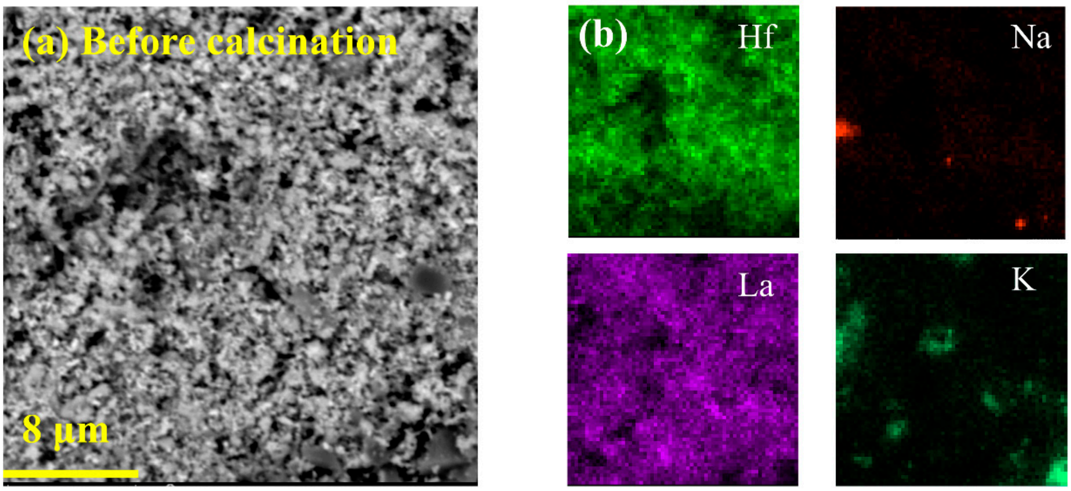

Figure S6. (a) SEM images of the fracture surfaces of the block sample (Φ20 mm, mass ratio of salt to reactants was 1:1) before calcination, and (b) the corresponding EDS-mapping.

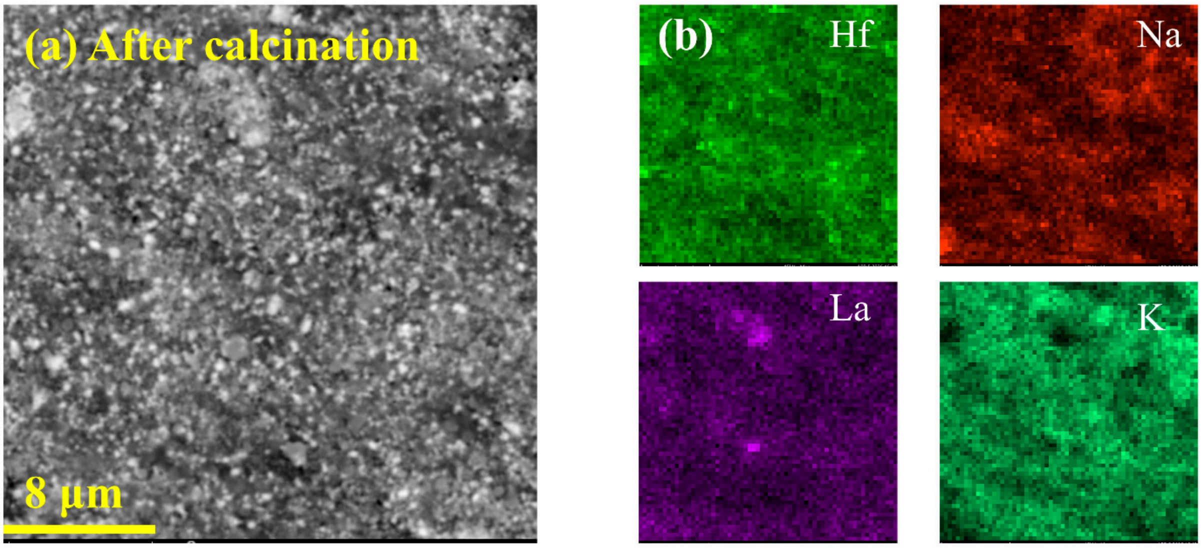

Figure S7. (a) SEM images of the fracture surfaces of the block sample (Φ20 mm, mass ratio of salt to reactants was 1:1) after calcination, and (b) the corresponding EDS-mapping.
